# Promotion of HepG2 cell apoptosis by *Sedum emarginatum* Migo and the mechanism of action

**DOI:** 10.1186/s12906-022-03503-6

**Published:** 2022-01-31

**Authors:** Qin Qiu, Lujuan Jiang, Hanshen Zhen, Fengyin Huang, Dandan Zhen, Meifang Ye, Xueyan Meng, Yuanyuan Liu, Xijun Qin

**Affiliations:** 1 Guangxi University of Chinese Medicine, Nanning, 530001 China; 2 Guangxi Superior Chinese Patent Medicine and National Medicine Development Engineering Technology Research Center, Nanning, 530001 China

**Keywords:** *S. emarginatum*, Ethyl acetate extract, HepG2, Apoptosis

## Abstract

**Background:**

*Sedum emarginatum* Migo(*S. emarginatum*) has anti-tumor and anti-oxidant effects. This study aimed to screen the extractions of *S. emarginatum* against liver cancer in vitro and explore its anti-liver cancer mechanism.

**Methods:**

The CCK-8(Cell Counting Kit-8) method was used to detect the inhibitory effect of different extracts of *S. emarginatum* on the proliferation of liver cancer HepG2 cells. The morphological changes of the cells after administration were observed with microscopy, cell apoptosis was detected by flow cytometry, and the expression of Bax, Bcl-2 and Caspase-3 mRNA in the cells were detected by RT-PCR (Reverse Transcription-Polymerase Chain Reaction) to explore the mechanism of action.

**Results:**

CCK-8 method test results showed that among the different extracts of *S. emarginatum*, the ethyl acetate extract(1000 μg/ml, 2000 μg/ml, 2500 μg/ml, 3000 μg/ml) and n-butanol extract(1000 μg/ml, 2000 μg/ml, 2500 μg/ml, 3000 μg/ml) have the strongest inhibitory effect on the proliferation of HepG2 cells. In these 4 concentrations, the inhibitory effect increased as the concentration increased. The IC_50_ of the ethyl acetate extract on HepG2 cells was less than that of the n-butanol extract, so the ethyl acetate extract has a better proliferation inhibitory effect on HepG2 cells than the n-butanol extract, followed by the 70% ethanol extract(3000 μg/ml) and the water extract(3000 μg/ml), petroleum ether extract was the weakest. The results of microscopy showed that ethyl acetate extract caused hepatocarcinoma HepG2 cell morphology changed, cell density decreased, and suspension cells increased. Moreover, the results of flow cytometry showed that the ethyl acetate extract of S. *emarginatum* could induce HepG2 cell apoptosis at the concentrations of 2500μg/ml and 3000μg/ml. RT-PCR results showed that the expression of Bax mRNA was up-regulate by the middle(2500 μg/ml) and high(3000 μg/ml) dose groups of ethyl acetate extract. The expression of Caspase-3 mRNA was up-regulated by the low(2000 μg/ml), medium(2500 μg/ml) and high(3000 μg/ml) dose groups of ethyl acetate extract. The expression of Bcl-2 mRNA was down-regulated by the high(3000 μg/ml) dose group of ethyl acetate extract.

**Conclusion:**

The ethyl acetate extract of *S. emarginatum* has the best effect on human liver cancer HepG2 cells. Its anti-hepatocellular mechanism may be related to affect the expression of apoptosis genes (Bax, Bcl-2 and Caspase-3mRNA) and promote the apoptosis of liver cancer cells. It provided a reference for the research and development of drugs for the treatment of liver cancer.

## Background

Cancer, as a complex disease that results from genetic and epigenetic modifications of tumour suppressor genes or oncogenes, can be developed because of alterations of apoptosis-signalling pathways [1]. Breakdown of the apoptosis process is observed in many human tumours, which may lead to transformation of a normal cell to a tumour cell [2]. Liver cancer is a common malignant tumor of the digestive system, and it is the fourth type of cancer-related cause of death after lung cancer, colon cancer and gastric cancer [3]. More than one-fifth of the population in China suffers from this disease [4]. According to the report of the International Cancer Organization, the incidence of liver cancer in China is the seventh in the world [5]. The mortality rate of liver cancer is greatly affected by the aging of the population. In the future, our country may experience an increase in the incidence of liver cancer due to the increase in the elderly population [6, 7]. At present, surgical resection combined with chemotherapy and radiotherapy is the main way to treat liver cancer. Although it reduces the incidence and mortality of liver cancer to a certain extent, it still faces the problem of poor prognosis for liver cancer patients [8–10]. Therefore, the search of highly effective and low-toxic anticancer drugs is a research hotspot.

In recent years, it has been discovered that traditional Chinese medicine has an interventional effect on tumors. Many components of traditional Chinese medicine have a significant inhibitory effect on different types of tumors, with fewer adverse reactions and good prognosis [11–15]. In addition, studies have shown that Crassulaceae plants have anti-tumor, immune regulation, and anti-aging effects [16–19]. *S. emarginatum* is one of the species of the Crassulaceae plants. It is widely distributed in China. Studies have shown that *S. emarginatum* may contain flavonoids, polysaccharides or glycosides, cardiac glycosides, steroids or triterpenes, and tannins, organic acids and other chemical components have anti-tumor and anti-oxidant effects, but their mechanism of action is not yet clear [20–24]. In recent years, the research on *S. emarginatum* has mainly focused on cultivation [25]. In addition, it also involves the analysis of UV-Visible Spectra [26] and content determination [27] of *S. emarginatum*, and the pharmacological research of the total flavonoids [28] of *S. emarginatum*. The latest published article related to *S. emarginatum* was: The complete chloroplast genome of Sedum emarginatum (Crassulaceae) [29]. At present, there is no public report on the promotion of HepG2 cell apoptosis by *S. emarginatum* and the mechanism of action.

Apoptosis is one of the primary targets for most conventional anti-cancer drugs [1]. Apoptosis, a programmed cell suicide, is usually a physiological event that does not induce inflammation [30]. Therefore, apoptosis induction is considered a desired therapeutic goal in cancer treatment to reduce possible adverse side effects [31]. In this study, the model cell used in liver cancer is the human liver cancer cell line HepG2 in the immortal cell line. This model cell has the advantages of stable growth, unlimited life span and stable phenotype, and is easy to standardize among different laboratories [32]. In this study, ethyl acetate extract, n-butanol extract, 70% ethanol extract, water extract, petroleum ether extract were obtained from *S. emarginatum*, and promotion of HepG2 cell apoptosis by *S. emarginatum* and the mechanism of action was studied. It fills the gap in the research on the promotion of hepatocarcinoma cell (HepG2) apoptosis by *S. emarginatum* and the mechanism of action. Provided a scientific basis for the further research of *S. emarginatum* as an anticancer drug.

## Materials and methods

### Experimental drugs and reagents

DMEM high glucose medium (lot number: 201809) was purchased from gibco; Sijiqing fetal bovine serum (lot number: 20180325) was purchased from Hangzhou Tianhang Biotechnology Co., Ltd.; (PBS) Phosphate buffer (batch number: 20180928), penicillin-streptomycin mixed solution (batch number: 201803), 0.25% trypsin digestion solution (batch number: 201710), EDTA-free trypsin digestion solution (lot number: 201901) were purchased from Solarbio, USA; Enhanced CCK-8 reagent was purchased from Shanghai Shangbao Biotechnology Co., Ltd.; (DMSO) dimethyl sulfoxide (lot number: EZ1609C224) was purchased from Biofroxx; AnnexinV-FITC/PI apoptosis double staining kit (lot number: 20181217), 1 × Binding Buffer (lot number: 20190114) were purchased from Jiangsu KGI Bio; Homo-BAX-R, Homo-BAX-F, Homo-Bcl2-R, Homo-Bcl-2-F, Homo-CASP3-R, Homo-CASP3-F, Homo-ACTB-R, Homo-ACTB-F were purchased from Shanghai Shenggong Synthetic Company; RNA extraction kit (article number: 9767, batch number: 20181124), reverse transcription kit (article number: RR036A), TB Green Premix Taq (article number: RR820) were purchased from TAKARA; absolute ethanol (article number: 64–17-5) was purchased from Guangzhou Chemical Reagent Factory; 25 cm air-permeable culture flask, 96-well plate, 6-well plate, and cryotube were purchased from corning; 0.22 μM syringe filter was purchased from Millipore.

### Plant material

*S. emarginatum* was collected from Ninghai, Zhejiang in August, 2017. In accord with the IUCN Policy Statement on Research Involving Species at Risk of Extinction and the Convention on the Trade in Endangered Species of Wild Fauna and Flora, there are no restrictions for the collection of this species. The plant was identified by Ma Lifei Deputy Chief Pharmacist (Deputy Chief Pharmacist of Guangxi Yixin Pharmaceutical Co., Ltd.). The voucher specimens were deposited in the Department of Drug Analysis, Guangxi University of Chinese Medicine (No 2017-0802). Ten times the amount of 70% ethanol was used to soak 150 g of rough powder of *S. emarginatum* for 30 min, refluxed and extracted 3 times, refluxed for 2 h, 1.5 h, 1 h respectively, filtered with gauze, combined the extracts, and concentrated on a rotary evaporator under reduced pressure obtained a 70% ethanol extract concentrate; Then extracted with petroleum ether, ethyl acetate and n-butanol for 6–7 times respectively, and concentrated under reduced pressure into petroleum ether, ethyl acetate and n-butanol extracts. The above three extract solutions and the remaining water layer were volatilized into extracts through a water bath (below 60 °C). After drying under reduced pressure, dry extract samples of *S. emarginatum* petroleum ether, ethyl acetate, n-butanol and water was prepared. Precisely weighed 100 mg of the samples of different polar extracts of *S. emarginatum* (70% ethanol extract, petroleum ether extract, ethyl acetate extract, n-butanol extract, water extract), respectively. DMSO was added to help dissolve (including DMSO≤0.5%), diluted with complete medium to the corresponding drug concentration, ultrasound to help dissolve, sterilized through 0.22 microporous membrane, subpackaged, and stored at − 20 °C.

### Cell culture

The human liver cancer HepG2 cell lines were obtained from the Chinese Academy of Sciences and was stored in the nitrogen solution of the Science Experiment Center of Guangxi University of Chinese Medicine. The frozen HepG2 liver cancer cells were thawed at 37 °C water bath, resuscitated, transfered to a 15 ml centrifuge tube, centrifuged for 5 min (1000 rpm), the supernatant was discarded, 5 ml of the mixed solution was added to the centrifuge tube. The mixed solution was made of DMEM high-sugar medium, Sijiqing fetal bovine serum, antibiotics (penicillin and streptomycin) in a ratio of 10:2:1, transferred to a culture flask, and incubated in a 5% CO_2_, 37 °C incubator.

### Determination of cell proliferation inhibition rate by CCK-8 method

The CCK-8 method was used to determine the cell proliferation inhibition rate. 1.5 ml 0.25% EDTA-trypsin was added to HepG2 cells, placed in a cell incubator for 2 min, the trypsin was discarded, 5 ml complete culture medium was added, HepG2 cells were seeded at a concentration of 5 × 10^5^/ml into 96-well culture plates, 100 μL per well, and incubated it at 37 °C, 5% CO_2_ incubator for 24 h. The blank control group and the administration group were added with 100 μL of diluents of different polar extracts of *S. emarginatum* at different concentrations(70% ethanol extract, petroleum ether extract, ethyl acetate extract, n-butanol extract, and water extract) to make the final concentration of the different polar extracts of *S. emarginatum* be 0, 250, 500, 1000, 2000, 2500, 3000 μg/ml, each concentration was set 3 wells, shook and mixed gently, placed in a 37 °C, 5% CO_2_ incubator and continued incubating for 48 h. The old culture medium was discarded, each well was added 100 μL of PBS and rinsed twice, then each well was added 100 μL of complete culture medium (contained 10% enhanced CCK-8 solution), continued to incubate for 3-4 h. The OD value was measured at a test wavelength of 490 nm. The OD value of the experimental group and blank control group were used to determine the cytotoxicity of extract according to the following formula: cell inhibition rate = 1-(OD value of experimental group/OD value of blank control group) × 100%.

### Microscopic examination

The HepG2 cells in the logarithmic growth phase were operated as above method, and after being treated with different concentrations of *S. emarginatum* ethyl acetate extract (0 μg/ml、250 μg/ml、500 μg/ml、1000 μg/ml、2000 μg/ml、2500 μg/ml、3000 μg/ml). HepG2 cells morphological apoptotic changes were examined using microscope (Olympus, CKX4, Japan).

### Flow cytometry to detect apoptosis

HepG2 cells were seeded at a concentration of 1 × 10^7^/ml into 6-well culture plates, incubated for 24 h, the culture medium was discarded. In the blank control group, only new complete culture medium was added, and the treatment group was added with 2000 μg/ml, 2500 μg/ml, and 3000 μg/ml concentrations of culture medium contained the ethyl acetate extract of *S. emarginatum*, after incubating for 48 h, cells were collected by trypsinization without EDTA to prepare a single cell suspension, centrifuged at 1000 r/min for 5 min, and the supernatant was discarded. About 1 ml of 4 °C precooled PBS was added, resuspended the cells. Centrifuged again to pellet the cells, supernatant was aspirated, diluted the binding buffer 1:3 with deionized water (4 ml 4 × binding buffer+ 12 ml deionized water), then resuspended the cells with 1 × binding buffer to adjust the concentration to 1–5 × 10^7^/ml, then 100 μL of cell suspension was taken into a 5 ml flow tube. The blank control group cells were not treated with AnnexinV and propidium iodide solution (PI). Five microliters of AnnexinV was added to the administration group and mixed well. After incubating at room temperature for 5 min in the dark, 10 μL 20μg/ml propidium iodide solution (PI) and 400 μl PBS were added, and flow cytometry was performed immediately (Millipore, Guava easyCyte HT, France). The experiment was repeated 3 times.

### RT-PCR detection

HepG2 cells were seeded at a concentration of 1 × 10^7^/ml into 6-well culture plates, incubated for 24 h, the culture medium was discarded. In the blank control group, only a new complete culture medium was added. The drug-treated group was added with different concentrations (2000 μg/ml, 2500 μg/ml, and 3000 μg/ml) of the medicated culture medium of the ethyl acetate extract of *S. emarginatum*. The cells were treated for 48 h. The experiment was repeated 3 times. After 48 h, the cells were collected, and the RNA was extracted by Trizol. The cDNA was reversed according to the TAKARA and RR036A kits, and then 2 × Master, Mix Forward Primer (10 μM), Reverse Primer (10 μM), Water, nuclease-free etal reagents was added, PCR amplification was performed (Agilent, AriaMx, USA). homo-BAX-F: CGGGTTGTCGCCCTTTTCTA, homo-BAX-R:GGAGACAGGGACATCAGTCG; homo-Bcl2-F: CTTTGAGTTCGGTGGGGTCA, homo-Bcl2-R:GGGCCGTACAGTTCCACAAA; homo-Caspase3-F: CGGCGCTCTGGTTTTCGTTA, homo-Caspase3-R: CAGAGTCCATTGATTCGCTTCC. Reaction conditions: 95 °C predenaturation for 30s, 95 °C for 5 s, 60 °C for 30s, 72 °C for 15 s, a total of 40 cycles, 95 °C for 15 s extension.

### Statistical analysis

All data were expressed as means±standard deviations, using SPSS 21.0 software for t-test, GraphPad for collating and mapping. Statistically significant differences were recorded as: *P < 0.05, and extremely significant differences were recorded as: **P < 0.01.

## Results

### Effects of different extracts of *S. emarginatum* to inhibit the proliferation of HepG2 cells

**Fig. 1:**
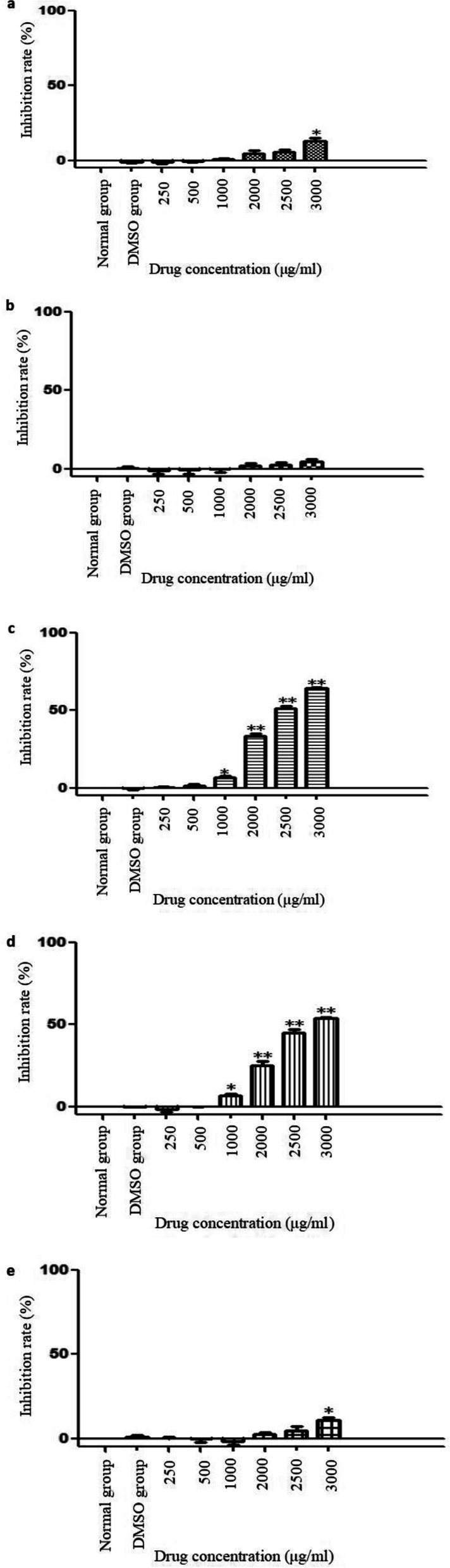
The CCK-8 method was used to screen the anti-hepatocarcinoma active extracts of *S. emarginatum*. The effect of the **a** 70% ethanol extract, **b** the petroleum ether extract, **c** the ethyl acetate extract, **d** the n-butanol extract, and **e** the water extract of *S. emarginatum* on the inhibition rate of HepG2 cells. *signified (*p* < 0.05) compared to the control, **signified (*p* < 0.01) compared to the control

The CCK-8 method was used to screen the anti-hepatocarcinoma active extracts of *S. emarginatum*. The results showed that when the petroleum ether extract of *S. emarginatum* was in the range of 0 ~ 3000 μg/ml, the 70% ethanol extract and the water extract were in the concentration range of 0 ~ 2500 μg/ml, the inhibitory effect on the proliferation of liver cancer HepG2 cells was not obvious, compared with the control group, there were no significant difference (*P* > 0.05); The 70% ethanol extract and water extract at 3000 μg/ml have inhibitory effect on the proliferation of liver cancer HepG2 cells (*P* < 0.05); compared with the control group, there were no significant difference between the ethyl acetate extract and the n-butanol extract at the concentrations of 250 μg/ml and 500 μg/ml (P> 0.05); compared with the control group, there were significant differences at the concentrations of 1000 μg/ml, 2000 μg/ml, 2500 μg/ml, 3000 μg/ml, (*P*<0.05 or *P*<0.01), these results indicated that the ethyl acetate extract and n-butanol extract of *S. emarginatum* could inhibit the proliferation of HepG2 cells, and in these 4 concentrations, the inhibitory effect increased as the concentration increased. According to the calculation results of SPSS software, the IC_50_ of the ethyl acetate extract and n-butanol extract of *S. emarginatum* against liver cancer HepG2 cells were 2528.08 μg/ml and 2783.37 μg/ml, respectively. Therefore, the inhibitory effect of ethyl acetate extract on the proliferation of human liver cancer HepG2 cells was better than that of n-butanol extract. The results were shown in Fig. 1 .

### Morphological changes observed under microscope

**Fig. 2:**
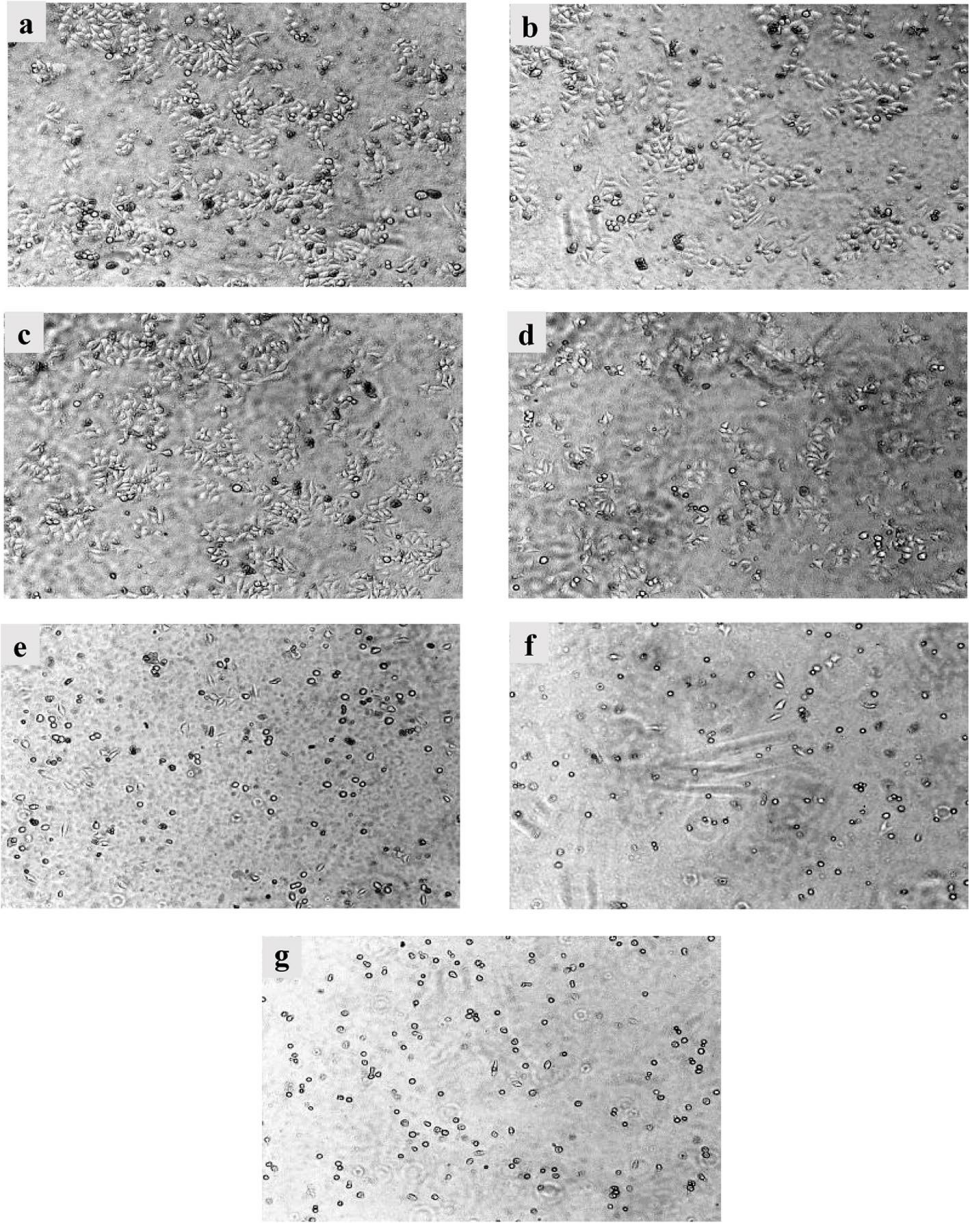
Effects of ethyl acetate extract of *S. emarginatum* on the morphology of HepG2 cells. Photomicrographs of HepG2 cells (**a**) no treatment and treatment with (**b**-**g**) 250 μg/ml, 500 μg/ml, 1000 μg/ml, 2000 μg/ml, 2500 μg/ml, 3000 μg/ml of the ethyl acetate extract of *S. emarginatum*

After applying the ethyl acetate extract of *S. emarginatum* to the liver cancer HepG2 cells for 48 h, the changes in cell morphology after administration could be observed. The cells in the blank control group and the 250 μg/ml and 500 μg/ml cells in the administration groups still adhered to the wall, showing irregular long spindle shapes, clear outlines and high cell density. Cells with administration concentrations of 1000 μg/ml, 2000 μg/ml, 2500 μg/ml, and 3000 μg/ml appeared in a state of spliting, rounding, and shedding suspension, the cell density decreased, and the suspended cells increased. The results were shown in Fig. 2.

### Effect of ethyl acetate extract of *S. emarginatum* on HepG2 cell apoptosis

**Fig. 3:**
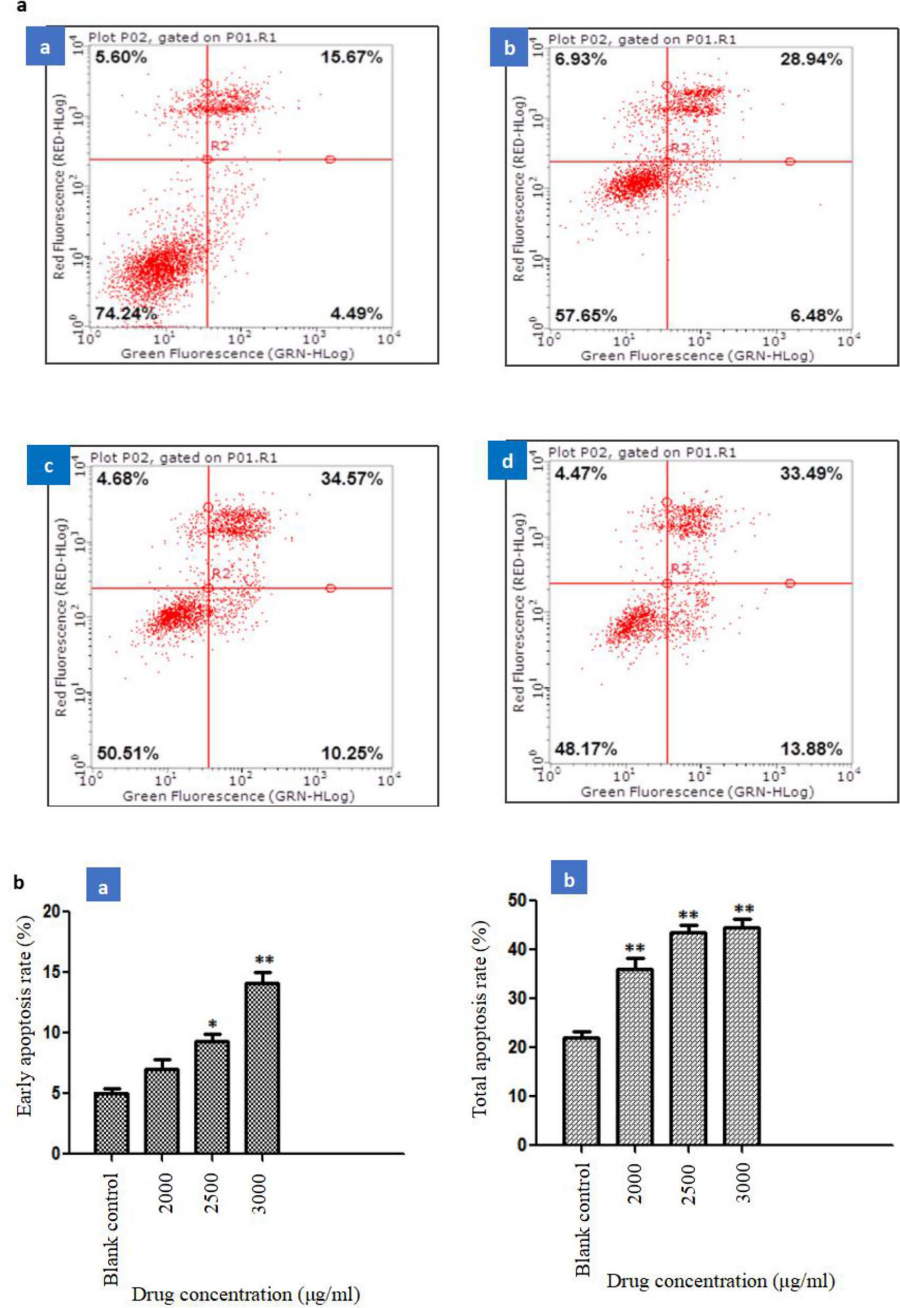
Effects of different concentrations of *S. emarginatum* ethyl acetate extract on HepG2 cell apoptosis. **a** Flow cytometry was used to detect the apoptosis of HepG2 cells (a A) untreated and treated with (a B)2000 μg/ml, (a C)2500 μg/ml, (a D)3000 μg/ml ethyl acetate extract of *S. emarginatum*. **b** The (b A) early apoptosis rate and (b B) total apoptosis rate of HepG2 cells no treatment and treatment with 3 doses of ethyl acetate extract for 48 h. Compared with the blank control group, * means *P*<0.05; Compared with the blank control group, ** means *P*<0.01

According to the results of the CCK-8 method to screen the active extracts of *S. emarginatum* against liver cancer, three doses of 2000 μg/ml, 2500 μg/ml, and 3000 μg/ml of the ethyl acetate extract of *S. emarginatum* were selected for AnnexinV-PI double staining. Flow cytometry was used to detect the apoptosis of HepG2 cells, and a blank control group was set up. It could be seen from the results of cell apoptosis detected by the flow cytometer in Fig. 3, the blank control group and the ethyl acetate extract with different administration concentrations acted on HepG2 cells for 48 h. The early apoptosis rate and the total apoptosis rate showed different degrees of apoptosis, and both increased with the increase of drug concentration. Compared with the blank control group, the early apoptosis rate of 2500 μg/ml, 3000 μg/ml concentrations (*P*<0.05 or *P*<0.01), the total apoptosis rate of 2000 μg/ml, 2500 μg/ml, 3000 μg/ml concentrations (*P*<0.01)), the results showed that the ethyl acetate extract of *S. emarginatum* could induce cell apoptosis.

### Effect of ethyl acetate extract of *S. emarginatum* on HepG2 cell apoptosis level

**Fig. 4:**
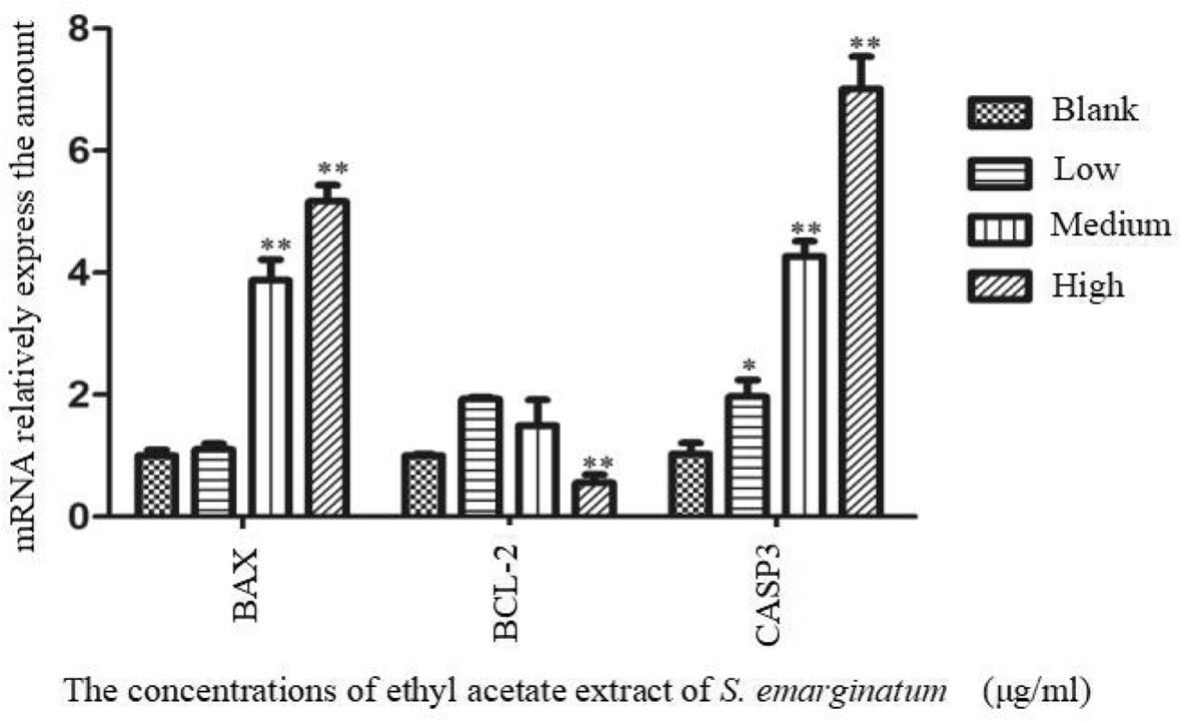
Effects of ethyl acetate extract of *S. emarginatum* on the expression of Bax, bcl-2 and caspase-3 mRNA in HepG2 cells. Compared with the blank control group, * means *P*<0.05; Compared with the blank control group, ** means *P*<0.01

Three doses of 2000 μg/ml, 2500 μg/ml, and 3000 μg/ml of ethyl acetate extract in *S. emarginatum* were selected for RT-PCR experiments, 2000 μg/ml, 2500 μg/ml, and 3000 μg/ml as low, medium and high dose groups respectively, and a blank control group was set up. RT-PCR was used to detect the effect of the ethyl acetate extract of *S. emarginatum* on the mRNA levels of Bax, Bcl-2 and Caspase-3 in HepG2 cells. The results were shown in Fig. 4. Compared with the blank control group, the middle and high-dose groups could up-regulate the expression of Bax mRNA (*P*<0.01), The low, middle, and high-dose groups could up-regulate the expression of Caspase-3 mRNA (*P*<0.05 or *P* < 0.01), and the high-dose group could down-regulate the expression of Bcl-2 mRNA (*P* < 0.01). The results showed that the ethyl acetate extract of *S. emarginatum* could promote the apoptosis of HepG2 cells.

## Discussion

In recent years, it has been found that *S. emarginatum* can inhibit the proliferation of lung cancer, oral cancer, esophageal cancer, liver cancer and other tumor cells, indicating that *S. emarginatum* has good application prospects in the treatment of liver cancer. However, the mechanism of *S. emarginatum* on the apoptosis of liver cancer HepG2 cells is still unclear. This study confirms the idea that *S. emarginatum* can inhibit the proliferation of liver cancer cells. According to related studies, the anti-tumor mechanism of traditional Chinese medicine is mainly divided into several types: inhibiting the proliferation of liver cancer cells, promoting apoptosis, reversing drug resistance, inhibiting cancer cell invasion and adhesion, inhibiting cancer cell migration, and inducing cancer cell apoptosis, etc. [33–35]. In this study, the CCK-8 method was used to screen then active extracts of anti-hepatocarcinoma in different polar extracts of *S. emarginatum*. The results showed that among the different polar extracts (70% ethanol extract, petroleum ether extract, ethyl acetate extract, n-butanol extract, water extract) of *S. emarginatum*, the ethyl acetate extract and n-butanol extract have the strongest inhibitory effect on the proliferation of HepG2 cells. Among them, the IC_50_ of the ethyl acetate extract on HepG2 cells was less than that of the n-butanol extract, so the ethyl acetate extract has a better proliferation inhibitory effect on HepG2 cells than the n-butanol extract, followed by the 70% ethanol extract and the water extract, petroleum ether extract was the weakest. Observation of cell morphology showed that different concentrations of *S. emarginatum* ethyl acetate extract could changed the morphology of liver cancer HepG2 cells. The cells appeared to split, round, and fall off and suspended. The morphology was irregular polygonal, and the nucleolus was concentrated or split, indicating ethyl acetate extract of *S. emarginatum* inhibited the growth of HepG2 cells. The results of flow cytometry showed that after different concentrations of the ethyl acetate extract of *S. emarginatum* treated liver cancer HepG2 cells for 48 h, HepG2 cells showed different degrees of apoptosis. Compared with the control group, the ethyl acetate extract of *S. emarginatum* had a significant apoptotic effect. The early apoptosis rate and total apoptosis rate increased with the increase of drug concentration, indicating that the ethyl acetate extract of *S. emarginatum* could induce apoptosis of HepG2 cells. Apoptosis is the main mechanism that inhibits the occurrence of malignant tumors, and its process includes gene activation, expression and regulation, etc. Promoting apoptosis can inhibit the growth of tumor cells. Therefore, inducing tumor cell apoptosis is an important way for tumor prevention and treatment [36]. Among them, apoptosis-related genes Bcl-2 and Bax belong to the Bcl-2 family members. Bcl-2 can inhibit cell apoptosis and has a protective effect on cells. Bax has the effect of promoting cell apoptosis. In tumor cells, the expression of Bax protein is often suppressed. When drugs or other means increase the expression of Bax, it can increase tumor cell apoptosis [37]. The Bcl-2 family is one of the important factors that regulate cell apoptosis, and the main site of action is mitochondria [38]. Among them, Bax, which is located in the cytoplasm, is a pro-apoptotic protein that has been studied extensively. When the cell receives a signal stimulus such as death, Bax translocates to the mitochondria. Bax not only interacts with the anti-apoptotic factor Bcl-2 on the mitochondrial membrane, but also interacts with the pro-apoptotic factor Apaf-1 in the mitochondria to inactivate the anti-apoptotic factor and increase the release of the pro-apoptotic factor. Destroy the structure and function of mitochondria, eventually activate the Caspase pathway and initiate apoptosis [39]. Caspase family proteases are the executors of cell apoptosis, which can induce cell apoptosis in a cascade way and directly lead to cell disintegration. Among them, Caspase-3 is an important apoptosis executive factor, which normally exists in the form of inactive proCaspase-3 zymogen. When stimulated by signals such as apoptosis, the zymogen is cleaved and activated. Activated cleaved Caspase-3 can inactivate and cleave DNA, repair related molecules, enzymatically hydrolyze the extracellular matrix, destroy cytoskeleton proteins, so that chromatin can accumulate, nucleus is broken, and cell morphology shrinks, and induces cells to enter irreversible programming death stage [40]. In order to further clarify the mechanism of the ethyl acetate extract of *S. emarginatum* in promoting cell apoptosis, RT-PCR was used to detect the effect of the ethyl acetate extract of *S. emarginatum* on the expression of Bcl-2 mRNA, Bax mRNA and Caspase-3 mRNA in liver cancer HepG2 cells. The results of the study showed that the ethyl acetate extract of *S. emarginatum* could up-regulate the expression of Bax mRNA (2500 μg/ml, 3000 μg/ml group) and Caspase-3mRNA (2000 μg/ml, 2500 μg/ml, 3000 μg/ml group) in HepG2 cells of liver cancer, and down-regulate the expression of Bcl-2 mRNA (3000 μg/ml group) in HepG2 cells. These results showed that the ethyl acetate extract of *S. emarginatum* could promote the apoptosis of liver cancer HepG2 cells by affecting the expression of related apoptosis genes.

## Conclusion

Taken together, in the vitro experiments of different polar extracts of *S. emarginatum*, ethyl acetate extract and n-butanol extract have the strongest inhibitory effect on the proliferation of liver cancer HepG2 cells. Among them, ethyl acetate extract has better inhibitory effect on HepG2 cell proliferation than n-butanol extract. Followed by the 70% ethanol extract and the water extract, and petroleum ether extract was the weakest. Our studies showed that *S. emarginatum* may regulated the expression of apoptosis genes such as Bcl-2 mRNA, Bax mRNA, and Caspase-3 mRNA to inhibit the proliferation of liver cancer HepG2 cells and promote cells apoptosis. This study showed that *S. emarginatum* is a promising drug for liver cancer treatment. Further in vivo experiments will improve the study results of the anti-liver cancer effect of *S. emarginatum*.

## Data Availability

All data generated or analyzed during this study are included in this published article.

## Abbreviations

CCK-8: Cell Counting Kit-8
RT-PCR: Reverse Transcription-Polymerase Chain Reaction
DMSO: Dimethyl sulfoxide
